# Visualization of Synchronous or Asynchronous Release of Single Synaptic Vesicle in Active-Zone-Like Membrane Formed on Neuroligin-Coated Glass Surface

**DOI:** 10.3389/fncel.2018.00140

**Published:** 2018-05-23

**Authors:** Junichiro Funahashi, Hiromitsu Tanaka, Tomoo Hirano

**Affiliations:** Department of Biophysics, Graduate School of Science, Kyoto University, Kyoto, Japan

**Keywords:** active zone, total internal reflection fluorescence microscopy, neuroligin, synaptic vesicle, synaptophysin, asynchronous release, exocytosis

## Abstract

Fast repetitive synaptic transmission depends on efficient exocytosis and retrieval of synaptic vesicles around a presynaptic active zone. However, the functional organization of an active zone and regulatory mechanisms of exocytosis, endocytosis and reconstruction of release-competent synaptic vesicles have not been fully elucidated. By developing a novel visualization method, we attempted to identify the location of exocytosis of a single synaptic vesicle within an active zone and examined movement of synaptic vesicle protein synaptophysin (Syp) after exocytosis. Using cultured hippocampal neurons, we induced formation of active-zone-like membranes (AZLMs) directly adjacent and parallel to a glass surface coated with neuroligin, and imaged Syp fused to super-ecliptic pHluorin (Syp-SEP) after its translocation to the plasma membrane from a synaptic vesicle using total internal reflection fluorescence microscopy (TIRFM). An AZLM showed characteristic molecular and functional properties of a presynaptic active zone. It contained active zone proteins, cytomatrix at the active zone-associated structural protein (CAST), Bassoon, Piccolo, Munc13 and RIM, and showed an increase in intracellular Ca^2+^ concentration upon electrical stimulation. In addition, single-pulse stimulation sometimes induced a transient increase of Syp-SEP signal followed by lateral spread in an AZLM, which was considered to reflect an exocytosis event of a single synaptic vesicle. The diffusion coefficient of Syp-SEP on the presynaptic plasma membrane after the membrane fusion was estimated to be 0.17–0.19 μm^2^/s, suggesting that Syp-SEP diffused without significant obstruction. Synchronous exocytosis just after the electrical stimulation tended to occur at multiple restricted sites within an AZLM, whereas locations of asynchronous release occurring later after the stimulation tended to be more scattered.

## Introduction

Synaptic vesicles are exocytosed at a presynaptic active zone immediately after arrival of an action potential, and their exocytosis can be repeated at high frequencies. Efficient retrieval mechanisms of release-competent synaptic vesicles support repetitive synaptic transmission (Haucke et al., 2011; Wu et al., 2014; Kononenko and Haucke, 2015). Previous studies have shown characteristics of exocytosis, endocytosis and synaptic vesicle retrieval using electrophysiological membrane capacitance measurement (von Gersdorff and Matthews, 1994; Yamashita et al., 2005; He et al., 2006) and fluorescence imaging (Miesenböck et al., 1998; Zenisek et al., 2000; Wienisch and Klingauf, 2006; Kavalali and Jorgensen, 2014). Most studies used repetitive activation of presynaptic neurons to improve the signal to noise ratio of data by increasing the total number of exocytosed synaptic vesicles, although some studies reported phenomena reflecting exocytosis of a single synaptic vesicle (Zenisek et al., 2000; Aravanis et al., 2003; Gandhi and Stevens, 2003; Balaji and Ryan, 2007; Chen et al., 2008; Zhang et al., 2009; Midorikawa and Sakaba, 2015; Tang et al., 2016; Maschi and Klyachko, 2017; Sakamoto et al., 2018).

The relative amounts of membrane proteins are presumed to be constant among synaptic vesicles in a presynaptic terminal (Takamori et al., 2006). After full-fusion exocytosis, synaptic vesicle proteins might remain clustered on the cell surface membrane so that they can be endocytosed as a package (Willig et al., 2006; Opazo et al., 2010), or they might be once dispersed and then gathered either on the cell membrane or on endosomal membrane to form release-competent vesicles (Wienisch and Klingauf, 2006; Gimber et al., 2015). However, the dynamics of synaptic vesicle proteins after a single exocytosis event have remained enigmatic, partly because of the limited spatiotemporal resolution of imaging data. Clarification of synaptic vesicle protein movement on the plasma membrane after exocytosis would provide critical information to reveal these dynamics.

Recently, the location of exocytosis of a synaptic vesicle has been studied intensively, and the existence of multiple release sites within an active zone has been suggested (Tang et al., 2016; Maschi and Klyachko, 2017; Miki et al., 2017; Sakamoto et al., 2018). Synaptic vesicle exocytosis occurs not only immediately after an action potential (synchronous release) but also tens or hundreds of msec after an action potential (asynchronous release). The Ca^2+^-dependence and molecular regulation mechanisms have been suggested to differ between the synchronous and asynchronous releases (Bacaj et al., 2013; Kaeser and Regehr, 2014). Relatively slow Ca^2+^ chelator EGTA preferentially suppresses asynchronous release but not synchronous release, suggesting that asynchronous release locations are more distant from the Ca^2+^ source than synchronous release sites. Thus, synchronous and asynchronous release locations could be differentially distributed within an active zone, but this has not been directly demonstrated.

We have addressed these unsettled questions about presynaptic mechanisms by developing a new visualization method with a high signal to noise ratio and high spatiotemporal resolution using total internal reflection fluorescence microscopy (TIRFM). We previously developed a method to induce formation of postsynaptic-like membrane on a glass surface coated with presynaptic cell-adhesion molecule neurexin, and analyzed exocytosis and endocytosis of glutamate receptor tagged with super-ecliptic pHluorin (SEP, a pH-sensitive variant of green fluorescent protein, Miesenböck et al., 1998) around the postsynaptic-like membrane (Tanaka and Hirano, 2012; Tanaka et al., 2014; Fujii et al., 2017). These studies prompted us to apply a similar method for analyses of presynaptic vesicle exocytosis. Presynaptic neurexin binds to postsynaptic neuroligin, and the interaction is involved in synaptic formation, maturation and/or maintenance. It has been demonstrated that the presence of neurexin or neuroligin on non-neuronal cell membrane can induce postsynaptic or presynaptic membrane differentiation in a nearby neurite, respectively (Scheiffele et al., 2000; Graf et al., 2004). Here, we report formation of a presynaptic active-zone-like membrane (AZLM) on a glass surface coated with neuroligin in a hippocampal neuronal culture preparation, and then show results of fluorescence imaging studies on the dynamics of a synaptic vesicle protein synaptophysin (Syp) after a single exocytosis event, and on the locations of synchronous and asynchronous releases in an AZLM.

## Materials and Methods

### Plasmids

Expression vectors of neuroligin-Fc and HA-neurexin were prepared as described previously (Scheiffele et al., 2000; Chih et al., 2006; Pettem et al., 2013) with minor modifications. Complementary DNA encoding the extracellular region (amino acids 1–675) of neuroligin 1 (−A +B) was fused to the N-terminal of human immunoglobulin-Fc region in pCAGplayII expression vector (Kawaguchi and Hirano, 2006). Neurexin 1β (−4) cDNA without its signal sequence (amino acids 1–47) was fused to the C-terminal of HA tag following a signal sequence in pDisplay expression vector (Invitrogen). An expression vector of EGFP was obtained from Clontech. EGFP-cytomatrix at the active zone-associated structural protein (CAST) was a gift from Dr. T. Ohtsuka (Yamanashi Univ.), and CAST was inserted into pTagRFPt-C vector (Shaner et al., 2008) modified from pTagRFP-C vector (Evrogen) to form TagRFPt-tagged CAST (CAST-RFP) expression vector. GCaMP6f was a gift from Dr. D.S. Kim (Chen et al., 2013; Addgene plasmid #40755). The expression vector of Syp-SEP was prepared as described previously (Granseth et al., 2006) with the following modification. The enhancer region of the CMV promoter was truncated (base pairs −300 to −67 relative to the transcription start site (+1) were deleted; Isomura et al., 2004) to reduce the expression level.

### Coating of Glass With Neuroligin

Glass coverslips coated with Fc-tagged neuroligin were prepared using methods similar to those in previous studies (Tanaka and Hirano, 2012; Tanaka et al., 2014), except that neuroligin-Fc was used instead of neurexin-Fc. In brief, neuroligin-Fc was prepared from transfected HEK293 cells (RRID:CVCL_0045) using nProtein A Sepharose (GE Healthcare). Glass coverslips were incubated with 43 μM biotinylated BSA (Thermo Scientific) in buffer A containing 100 mM KCl, 5 mM MgCl_2_, 25 mM HEPES, pH adjusted to 7.4 with KOH overnight at 4°C, followed by incubation with 17 μM streptavidin (WAKO) in buffer A for 1 h at room temperature (20–25°C). The coverslips were then incubated with biotin-conjugated anti-human IgG (1:100, Jackson ImmunoResearch) in buffer A for 1 h, followed by incubation with 3–5 μg/ml neuroligin-Fc in buffer A for 5 h. The coverslips were washed three times with buffer A after each incubation. Finally, the coverslips were incubated with 0.2 mg/ml poly-D-lysine (Sigma) for 12–48 h at 37°C.

### Primary Neuronal Culture and Transfection

All experimental procedures were carried out in accordance with guidelines laid down by the National Institutes of Health of USA and Kyoto University, and approved by the local committee for handling experimental animals in the Graduate School of Science, Kyoto University.

Hippocampal neuronal cultures were prepared from E18–20 Wistar rats (RRID:RGD_13508588) and grown on neuroligin-Fc-coated coverslips using methods similar to those described in a previous report except that Neurobasal-Electro rather than Neurobasal was used as the culture medium (Tanaka and Hirano, 2012). Lipofectamine 2000 (Invitrogen) was used to transfect plasmids into neurons at 9–10 DIV, and all experiments were performed 2 days after transfection.

### Immunocytochemistry

Cultured neurons were fixed with 4% paraformaldehyde and 4% sucrose in phosphate buffered saline (PBS) for 5 min at room temperature, and washed with PBS three times. Neurons were permeabilized and non-specific binding of antibodies was blocked with 0.2% Triton X-100 and 5% goat serum in PBS, respectively. Then, the preparation was incubated with primary antibody overnight at 4°C, and with secondary antibody for 1 h at room temperature. Images were acquired using TIRFM. Antibodies used here were anti-Bassoon (1:1000, Enzo; RRID:AB_10618753); anti-Piccolo (1:1000, Synaptic Systems; RRID:AB_887759); anti-Munc13 (1:1000, Synaptic Systems; RRID:AB_887733); anti-RIM (RAB3A-interacting molecule) 1/2 (1:1000, Synaptic Systems; RRID:AB_887775); goat anti-mouse or goat anti-rabbit IgG conjugated with Alexa 488 (Invitrogen; RRID:AB_138404 or AB_2576217, respectively).

### Live-Cell Imaging With TIRFM

TIRFM imaging was performed using an inverted fluorescence microscopy IX71 (Olympus) equipped with ×150/1.45 TIRFM objective lens (Olympus), ×1.6 intermediate lens (Olympus), 488 nm laser (85-BCD-020, Melles Griot, Albuquerque, NM), 561 nm laser (Sapphire 561LP, Coherent, Santa Clara, CA) and EM-CCD camera (iXon+; Andor), yielding a final effective pixel size of 67 nm. The electrical field stimulation (1 ms, 20–30 V/cm) was applied through platinum electrodes as described previously (Tanaka et al., 2014). All live-cell imaging experiments were performed at room temperature in extracellular imaging solution (119 mM NaCl, 2.5 mM KCl, 4 mM CaCl_2_, 1 mM MgCl_2_, 30 mM glucose, 25 mM HEPES; adjusted to pH 7.4 with NaOH). The solution also contained 10 μM 6-cyano-7-nitroquinoxaline-2,3-dione (CNQX) to inhibit AMPA-type glutamate receptor.

### Ca^2+^ Imaging

For Ca^2+^ imaging, neurons were transfected with GCaMP6f (Chen et al., 2013), CAST-RFP and HA-neurexin, and observed with TIRFM. CAST-RFP images were acquired in 35 × 35 pixels surrounding an AZLM, and the GCaMP signal change induced by 100 Hz, five electrical pulses stimulation was recorded for 1 s. An image was recorded for 35 ms exposure time, and acquisition of the following image started after 4 ms transfer time. GCaMP fluorescence change was analyzed using ImageJ (NIH). First, a baseline image was generated by averaging six images recorded before the stimulation, and this baseline was subtracted from each image. A region of interest (ROI, 15 × 15 pixels) was set around a CAST-positive active zone, and the average intensity in the ROI was determined.

### Imaging of Syp-SEP Before and After Exocytosis

To record exocytosis events and also to monitor synaptic vesicle protein dynamics, Syp-SEP (synaptophysin-pHluorin) was co-transfected to neurons with CAST-RFP and HA-neurexin. 50 Hz, five electrical pulses stimulation was applied first, and a region (35 × 35 pixels) surrounding a CAST-RFP-positive area showing a clear increase in the SEP signal intensity was selected. Then, 488 nm light was applied for 30 s to photo-bleach the extracellular Syp-SEP signal. The SEP signal change induced by single pulse stimulation was recorded 140–300 times in each AZLM. In each trial, an image was recorded for 35 ms exposure time, and after 4 ms transfer time the following image was acquired. Three and six images respectively were captured before and after the stimulation in each trial. We waited for 6 s before starting the next trial to reduce the probability of recording the bounce event, which reflects radial movement of an intracellular fluorescent vesicle presumably formed by endocytosis of previously exocytosed Syp-SEP (Midorikawa and Sakaba, 2015). After about 140 trials the probability of detecting a Syp-SEP signal increase immediately after the stimulation decreased in most cases. The data obtained after the decrease were excluded from analyses. We also recorded CAST-RFP images every 2–3 min in order to correct the drift of the preparation in the off-line analyses.

Syp-SEP fluorescence change after the single pulse stimulation was analyzed using custom routines of MATLAB (Mathworks; RRID:SCR_001622) as described previously (Tang et al., 2016) with some modifications. First, the base line image was obtained by averaging three images recorded before the stimulation. This averaged image was subtracted from each image recorded in a single trial to diminish background signal. Then, a 2-dimensional Gaussian function was used to fit signal intensities of 15 × 15 pixels surrounding the peak in the image recorded during 2–37 ms after the stimulation. If the fitted Gaussian function showed peak intensity > 100 a.u., and 0.4 < SD < 3 pixels, the trial was used for further analysis, and the 3 × 3 pixel area surrounding the fitted peak was selected as the ROI. When the total signal intensity of the ROI just after the stimulation was > the mean + 3 SD intensity of three baseline images recorded before the stimulation and also > 1000 a.u., we examined the time course of events. If the total Syp-SEP signal intensity of the ROI in the 2nd frame (41–76 ms) recorded after the stimulation was > 20% of that in the 1st frame (2–37 ms), we assumed that synchronous exocytosis occurred. We occasionally recorded an instantaneous Syp-SEP signal increase which showed a fast signal decrease (the intensity became < 20% in the next frame). We thought that most of these were bounce events of previously endocytosed vesicles (Midorikawa and Sakaba, 2015) and excluded them from data analyses. We thought that these were bounce events, because the frequency of their occurrence increased when the interval between trials was shorter, which should have increased the number of fluorescent intracellular vesicles containing endocytosed Syp-SEP. In four of the trials among all those performed in the experiments, the distribution of Syp-SEP signal intensity could be fitted with the sum of two Gaussian functions whose centers were > 300 nm apart. In these cases, we presumed that synchronous release occurred at two locations, and the data were excluded from analyses except for the count of the number of synchronous release events. Asynchronous exocytosis was analyzed similarly except that the peak timing of the event relative to the stimulation was different. We did not include data obtained from an AZLM showing < 15 synchronous release events for the analyses.

### Distribution of Exocytosis Locations

We assumed the center of the Gaussian fit of the Syp-SEP signal in 15 × 15 pixels in the image showing the highest intensity in a trial to be the location of exocytosis. To examine whether exocytosis locations were distributed randomly in an AZLM or not, we first determined an AZLM region with the intensity threshold of CAST-RFP signal obtained using the Otsu method (ImageJ, NIH). Next, Monte-Carlo simulation was performed to get random distribution of the same number of exocytosis locations in an AZLM, which was repeated 100 times. Then, the nearest neighbor distances (NND) were measured in both experimental and simulation data (Miki et al., 2017). The clustering tendency of synchronous release locations within an AZLM was evaluated using a previously established hierarchical clustering analysis with cluster diameter of 50 nm (Maschi and Klyachko, 2017). Then, the distance between each exocytosis location and the nearest center of a cluster was measured.

### Estimation of Syp-SEP Diffusion Coefficient

In order to estimate the diffusion coefficient of Syp-SEP, we performed simulation of particle movement as in a previous study (Sochacki et al., 2012) with MATLAB. Ten thousand particles were set randomly in an 80 nm-diameter circle, and each particle moved randomly to one of four directions, up, down, left, or right by √2DΔt in one step. D is the assumed diffusion coefficient and Δt is the step time (0.5 ms). Particle positions at each time were summed for 35 ms, which corresponded to each recording period. The simulation was performed with various values of D. Then, the time course of experimental Syp-SEP signal decay in 3 × 3 pixels was compared with the simulation results, and the diffusion coefficient was calculated with the least squares method. We also estimated the diffusion coefficient by comparing the signal distribution of experimental data recorded during 41–76 ms after the stimulation with the simulated signal distribution using the least squares method.

### Statistics

All values are presented as mean ± SEM unless otherwise stated. Statistical analyses were performed using MATLAB. The Mann-Whitney U-test was used to evaluate the significance of differences between 2 groups. The Wilcoxon signed-rank test or U-test with Bonferroni correction was used when means of more than two groups were compared.

## Results

### Formation of AZLM on the Glass Surface

**Figure 1:**
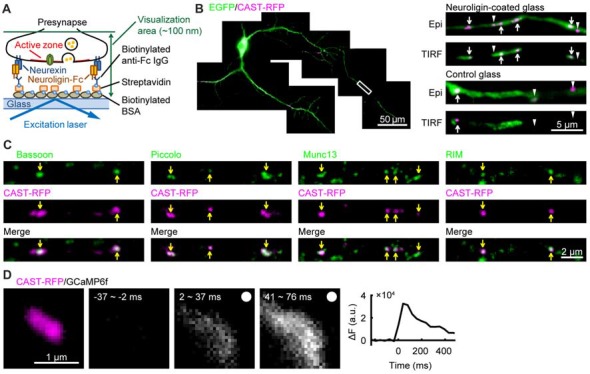
Formation of active-zone-like membranes (AZLMs) on neuroligin-coated glass. **(A)** Schematic presentation of AZLM formation on a cover glass coated with neuroligin. **(B)** AZLMs and normal active zones observed with an epi-fluorescence (Epi) or total internal reflection fluorescence (TIRF) condition. White rectangular area in the left image was magnified in the right top. Green and magenta show EGFP and cytomatrix at the active zone-associated structural protein (CAST)-RFP signals, respectively. **(C)** Co-localization of CAST-RFP (magenta) with Bassoon, Piccolo, Munc13 or RIM (green). **(D)** GCaMP6f signal increase around an AZLM marked with CAST-RFP (left magenta). The right graph shows the time course of change of GCaMP signal intensity. Electrical stimulation (100 Hz, 5 pulses) was applied at 0 ms.

A piece of cover glass was coated with the extracellular domain of neuroligin 1 using a method similar to that used to coat a glass surface with neurexin (Tanaka and Hirano, 2012; Tanaka et al., 2014), and rat hippocampal neurons were cultured on it (Figure 1A). A marker protein of presynaptic active zone CAST (Ohtsuka et al., 2002) fused to red fluorescent protein tag-RFPt (CAST-RFP) and EGFP were expressed in cultured hippocampal neurons. In neurons on the neuroligin-coated glass, CAST-RFP positive puncta were found on axons under conventional epi-fluorescence illumination, and some of them were also observed with total internal reflection fluorescence (TIRF) illumination, indicating that they were located very close to the glass surface. Most of such CAST-RFP signals were well observed in a single focusing plane just above the glass surface, suggesting parallel formation of CAST-positive areas on the neuroligin-coated glass. In this study we focused on such areas formed by excitatory neurons possessing spines on the dendrites. On the other hand, neurons cultured on the non-coated glass showed CAST-RFP positive puncta under epi-fluorescence, most of which were undetectable under TIRF illumination (Figure 1B). We next examined whether other presynaptic active-zone proteins were concentrated in CAST-RFP-positive active-zone-like structures. Active-zone proteins, Bassoon, Piccolo, Munc13 and RIM (Schoch and Gundelfinger, 2006; Südhof, 2012) were co-localized with CAST-RFP under TIRF illumination (Figure 1C). Each CAST-RFP-positive area was larger than the respective areas for the other presynaptic active-zone-proteins, a difference that might have been caused by overexpression of CAST-RFP. In addition, electrical field stimulation induced a transient increase in intracellular Ca^2+^ concentration in and around the CAST-RFP-positive area (Figure 1D). These results together suggest that a presynaptic active-zone-like membrane (AZLM) was formed directly on the neuroligin-coated glass.

### Detection of Single Synaptic Vesicle Exocytosis

**Figure 2:**
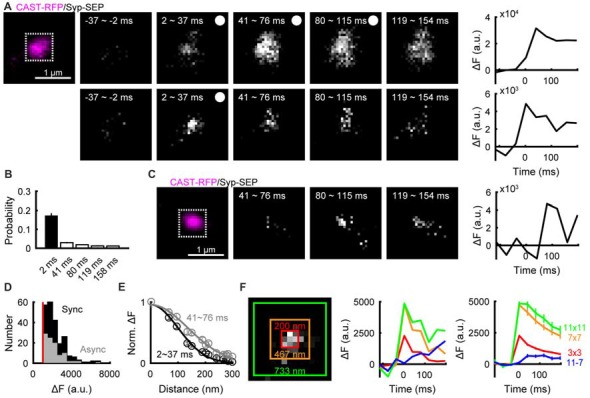
Detection of exocytosis of a single synaptic vesicle and diffusion of synaptophysin (Syp)-super-ecliptic pHluorin (SEP) in AZLMs. **(A)** Images showing Syp-SEP signal increase after stimulation with 5 pulses at 50 Hz (upper) or after a single pulse stimulation (lower) in an AZLM (left, magenta), and the time courses of Syp-SEP signal intensity in 11 × 11 pixel areas (white square in the left image) after five pulse stimulation or a single pulse stimulation. The signal intensity is enhanced in the lower images compared with the upper. **(B)** Graph showing occurrence probability of a release event induced by a single pulse stimulation in each frame (2–37, 41–76, 80–115, 119–154, 158–193 ms) per trial throughout all experiments (10 AZLMs). **(C)** Asynchronous release in an AZLM (left, magenta) after a single pulse stimulation and the time course of Syp-SEP signal intensity in 11 × 11 pixels (white square). **(D)** Amplitude histograms of synchronous (black) or asynchronous (gray) release events in 3 × 3 pixels. **(E)** Distributions of Syp-SEP signal intensity in each pixel plotted against the distance from the center pixel at 2–37 ms (black) and 41–76 ms (gray). These are averaged data obtained from all synchronous events in 10 AZLMs. **(F)** Time courses of Syp-SEP signal intensity in 3 × 3 (red), 7 × 7 (orange), 11 × 11 (yellow green) and 11 × 11–7 × 7 (blue) pixels surrounding the center. The left graph shows data obtained in one trial in an AZLM shown in the left image, and the right one shows the averaged data from all synchronous release events in 10 AZLMs. Means and SEMS are shown in the right graph.

We then examined whether synaptic vesicle exocytosis occurred in AZLMs. Exocytosis of synaptic vesicles containing Syp-SEP can be detected as an increase of fluorescence signal, because SEP, which is non-fluorescent in the low pH intra-vesicular solution, becomes fluorescent after exposure to the neutral pH of extracellular solution (Kavalali and Jorgensen, 2014). Repetitive electrical field stimulation (50 Hz, 5 pulses) clearly increased the intensity of the Syp-SEP signal in an AZLM (Figure 2A), indicating that synaptic vesicles were exocytosed in an AZLM. Next, we tried to detect a single exocytosis event by one pulse stimulation, which sometimes induced a transient increase of Syp-SEP signal followed by signal dispersal (Figure 2A). In 0 mM Ca^2+^ extracellular solution, such an increase of Syp-SEP signal was not recorded. The increase in Syp-SEP signal intensity occurred just after the electrical stimulation (synchronous release) in most cases (Figure 2B), although a slightly delayed signal increase (asynchronous release) was observed occasionally (Figures 2B,C). The asynchronous release event has been considered to reflect exocytosis of a single synaptic vesicle, and the signal intensities of asynchronous and synchronous events in 3 × 3 pixels were similar (synchronous, 2233 ± 72 a.u., *N* = 226 events; asynchronous, 2115 ± 94 a.u., *N* = 99 events; 10 AZLMs, *p* = 0.50, U-test; Figure 2D). Taking this finding together with the relatively low probability of occurrence of synchronous release (17 ± 1%, 226/1340 total trials in 10 AZLMs), we concluded that most synchronous events reflect single vesicle exocytosis, although there could be some events (<3%) reflecting release of multiple vesicles. We recorded simultaneous synchronous releases at two locations with a distance of >300 nm only four times throughout our experiments (1.7%). In these cases, we presumed that synchronous release occurred at two locations, and the data were excluded from analyses except for the count of the number of synchronous release events. We note that definition of synchronous release here is a significant increase of the Syp-SEP signal detected in the first frame (2–37 ms) after the stimulation. Thus, some release events which we assumed to be synchronous could have occurred later in the time frame, which corresponded to asynchronous releases by a conventional electrophysiological criterion (Kaeser and Regehr, 2014).

### Lateral Diffusion of Syp-SEP After a Single Exocytosis Event

In most cases, relatively slow decay of Syp-SEP signal intensity was observed after single pulse stimulation (Figure 2A), suggesting that full-fusion exocytosis rather than instantaneous kiss-and-run exocytosis (Aravanis et al., 2003; Zhang et al., 2009; Wu et al., 2014) took place. Next, we analyzed in detail how the Syp-SEP signal changed in a presumptive single exocytosis event. The center of the increased Syp-SEP signal was determined by fitting signal intensities with a two-dimensional Gaussian distribution in a frame recorded just after the electrical stimulation (2–37 ms; Figure 2E), and then the spatial distribution of the Syp-SEP signal was examined. The Syp-SEP signal recorded during 2–37 ms after the stimulation showed a larger peak and narrower distribution than that recorded during 41–76 ms, suggesting that lateral diffusion of Syp-SEP on the plasma membrane occurred after exocytosis (Figures 2A,E). Next, the total Syp-SEP signal intensities in 3 × 3, 7 × 7 and 11 × 11 pixels surrounding the center in the 2–37 ms image were measured in different time frames. After the increase in the 2–37 ms frame, the signal intensity decreased in all of the 3 × 3, 7 × 7 and 11 × 11 pixel areas, but the signal decay in 11 × 11 pixels was slowest (Figure 2F). In the 3 × 3, 7 × 7 and 11 × 11 pixel areas, the Syp-SEP signal intensity decreased to 35 ± 4%, 48 ± 5% and 56 ± 7%, respectively, of the peak value in the 197–232 ms frame. The decrease in the 11 × 11 pixel area was significantly slower than that in 7 × 7 pixels (*p* = 0.003, Wilcoxon signed-rank test with Bonferroni correction) or in 3 × 3 pixels (*p* < 0.001). The total signal intensity in 11 × 11 pixels minus that in 7 × 7 pixels increased with time (Figure 2F, blue line), supporting the conclusion that lateral spread of Syp-SEP occurred by diffusion.

Fitting the time course of the Syp-SEP signal decrease in 3 × 3 pixels surrounding the center to the simulation results with the least squares method gave an estimate of the diffusion coefficient of 0.17 ± 0.02 μm^2^/s (Figure 2F, *N* = 10 AZLMs). The diffusion coefficient was also estimated by comparing the lateral spread of the Syp-SEP signal in the 41–76 ms frame with the simulation data, which provided an estimate of 0.19 ± 0.02 μm^2^/s.

An instantaneous Syp-SEP signal increase whose intensity rapidly decayed to <20% in the next time frame occurred just after the stimulation in 3% of trials. Such signal increases were also recorded in the 41–76 ms or 80–115 ms frame in 1.6 or 1.5% of trials, respectively. We think that most of these were due to bounce events of previously endocytosed vesicles (Midorikawa and Sakaba, 2015), and we excluded them from data analyses. We think that these were due to bounce events, because their frequency increased when the interval between trials was shorter, a condition which should have increased the number of fluorescent intracellular vesicles containing endocytosed Syp-SEP.

### Distribution of Synchronous and Asynchronous Release Locations

**Figure 3:**
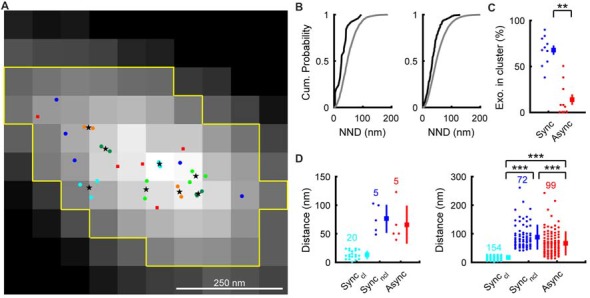
Distribution of synchronous and asynchronous release locations in an AZLM. **(A)** Distribution of synchronous and asynchronous release locations in a representative AZLM. The AZLM area defined using the Otsu method was marked with yellow lines. Synchronous release events are categorized as those that occurred in clusters (marked with light blue, yellow green, dark green or orange circles) and those that occurred outside of any clusters (dark blue circles). Centers of individual cluster are marked with black stars. Asynchronous release locations are marked with red squares. **(B)** Cumulative probability plots of the nearest neighbor distances (NND) between synchronous release locations (black) and those obtained by Monte-Carlo simulation assuming random distribution (gray). The left graph shows data obtained from the AZLM shown in **(A)**, and the right one shows averaged data from all AZLMs. **(C)** Ratios of synchronous (dark blue) or asynchronous (red) release events occurring in synchronous release clusters in each AZLM. Small circles represent percentages in respective AZLMs and a square and a bar represent mean and SEM. **p < 0.01, U-test. **(D)** Distribution of distances between each release location and the nearest center of a synchronous release cluster (light blue, synchronous release in a cluster; dark blue, synchronous release outside of any clusters; red, asynchronous release). The left graph shows data obtained from the AZLM shown in **(A)**, and the right one shows data from all AZLMs. Small circles represent individual data and a square and a bar represent mean and SD, respectively. Numbers in the graph indicate the numbers of respective release locations. ***p < 0.001, U-test with Bonferroni correction.

We assumed that the center of Gaussian fit in the Syp-SEP image showing the highest peak signal intensity was the location of exocytosis, in a similar manner to Tang et al. (2016). The estimated release locations were not distributed evenly, but rather were distributed in restricted regions within AZLMs (0.13–0.34 μm^2^; Figures 3A,B). We also noticed that synchronous release locations tended to form clusters. We then performed hierarchical clustering analysis of release locations with a diameter of 50 nm as reported previously (Maschi and Klyachko, 2017). There were 5.9 ± 0.6 (3–8) clusters of synchronous release locations which presumably corresponded to release sites (Maschi and Klyachko, 2017; Miki et al., 2017; Sakamoto et al., 2018), and 68 ± 5.0% (6/16–17/19, 154/226 locations in 10 AZLM) of synchronous releases occurred in the clusters (Figures 3C,D). On the other hand, only 14 ± 6% (0/12–2/4, 12/99 locations in total, *p* < 0.01, U-test) of asynchronous releases occurred in the clusters of synchronous release locations. Thus, synchronous release tended to occur repeatedly in multiple restricted release sites, as has been suggested (Zenisek et al., 2000; Tang et al., 2016; Maschi and Klyachko, 2017; Miki et al., 2017; Sakamoto et al., 2018), whereas asynchronous release tended to occur outside of such clusters. Supporting this idea, the shortest distance between the center of a synchronous release cluster and each synchronous release location (40 ± 3 nm, *N* = 226) was significantly smaller than that between the center of a synchronous release cluster and each asynchronous release location (68 ± 4 nm, *N* = 99, 10 AZLM, *p* < 0.001, U-test; Figure 3D). We would like to note that synchronous release locations outside of clusters were more distant from the nearest center of a cluster (90 ± 5 nm, *N* = 72) than asynchronous release locations (*p* < 0.001, U-test; Figure 3D). These results might be in line with an idea that some releases we regarded as synchronous might have occurred later in the 2–37 ms frame and could have been asynchronous events by an electrophysiological standard (Kaeser and Regehr, 2014).

## Discussion

Here, we detected exocytosis events of a single synaptic vesicle in AZLMs formed on neuroligin-coated glass and analyzed the dynamics of synaptic vesicle proteins upon exocytosis. Various presynaptic proteins were concentrated in an AZLM, and electrical stimulation induced intracellular Ca^2+^ increase and fast synaptic vesicle exocytosis. Thus, AZLMs were equipped with active-zone molecules and showed basic functions of active zones. However, we should note that an AZLM is an artificial structure, might be somewhat immature and/or might not show all normal functions of mature hippocampal synapses. The release probability (0.17 per AZLM) in 4 mM Ca^2+^ containing solution was lower than those reported previously. For example, Sakamoto et al. (2018) reported the release probability per release site was 0.51 in 4 mM Ca^2+^ containing solution. The apparent low release probability in AZLMs might have been caused partly by the procedure of pre-bleaching of cell surface Syp-SEP before recording, which might have eliminated some signals derived from recently endocytosed vesicles. Thus, the release probability could have been underestimated in this study. We also note that it was difficult to stably record exocytosis events >150 times. The vesicle retrieval mechanism might have been somewhat immature. Insufficient interaction of presynaptic proteins with postsynaptic adhesive proteins other than neuroligin 1, such as SynCAM, LRRTM or cadherin (Gerrow and El-Husseini, 2006), might have been a cause of immaturity. We also noticed that sizes of AZLMs were larger than those of normal active zones (Tang et al., 2016; Maschi and Klyachko, 2017; Sakamoto et al., 2018), and that synaptic vesicle release did not occur in some areas of AZLMs. Over-expression of CAST-RFP might have expanded CAST-RFP-positive areas in our preparation, and some parts might not have been well-organized as release-competent areas, possibly due to an insufficient amount of active-zone proteins there. Nevertheless, we think that application of TIFRM on AZLMs formed in a single focus plane located very close to the bottom glass surface, has provided high spatiotemporal resolution images of fluorescence-tagged synaptic-vesicle protein Syp-SEP, and allowed us to precisely analyze Syp-SEP dynamics and the distribution of both synchronous and asynchronous release locations in AZLMs.

After full-fusion exocytosis, synaptic vesicle proteins could be endocytosed as a package without dispersion (Opazo et al., 2010) or diffused on the surface membrane and then endocytosed (Gimber et al., 2015). Our results support the latter hypothesis and suggest that synaptic vesicular membrane proteins become scattered on the plasma membrane after membrane fusion, although only Syp was examined in this study. Whether other synaptic vesicle proteins such as synaptobrevin, synaptotagmin and/or vesicular glutamate transporter move similarly to Syp is an important question to be addressed in the future. The estimated diffusion coefficient of Syp-SEP (0.17–0.19 μm^2^/s) after single vesicle exocytosis was similar to the value reported in a previous study (Gimber et al., 2015) and to that of t-SNARE protein syntaxin (Ribrault et al., 2011), and was close to the fastest protein diffusion on the plasma membrane. Thus, it is suggested that synaptic vesicle protein Syp diffuses quickly on the plasma membrane upon vesicle exocytosis.

We found that locations of synchronous release of a synaptic vesicle tended to form clusters in an AZLM, and the number of such release sites was 3–8, which was similar to the number of release sites within an active zone reported in recent studies (Tang et al., 2016; Maschi and Klyachko, 2017; Sakamoto et al., 2018). Interestingly, we found that asynchronous release locations were distributed more widely than synchronous release locations. Thus, preferential synchronous and asynchronous release locations could be different. Zenisek et al. (2000) reported that synchronous releases tend to occur repeatedly at restricted sites and asynchronous releases do at isolated locations in a large presynaptic terminal of retinal bipolar cell. We found small fractions (32%) of synchronous release occurred outside of the release clusters. We might have failed to record repetitive synchronous releases in such locations because of the relatively small number of trials. Alternatively, such release events might not have been synchronous, but actually asynchronous ones, which occurred in the later time within the first frame (2–37 ms) after the stimulation. Asynchronous release might have occurred at locations not very close to Ca^2+^ channels (Miki et al., 2017). It was also reported that different subtypes of synaptotagmin (syt1 and syt7) are involved in synchronous and asynchronous releases (Bacaj et al., 2013; Kaeser and Regehr, 2014). Different locations for synchronous and asynchronous releases might reflect sub-active-zonal molecular organization such as distributions of different types of synaptotagmin-interacting proteins in AZLMs.

The visualization methods we have developed here could easily be applied to other synaptic proteins and would provide useful information about the dynamics of respective presynaptic proteins in the future. In addition, the combination of these methods with rapid extracellular pH change methods (Fujii et al., 2017) might be able to visualize endocytosis of synaptic vesicle proteins around AZLMs. Thus, we think that AZLMs will be useful for studying basic functional properties of presynaptic active-zones.

## Author Contributions

JF and TH contributed conception and design of the study and wrote the manuscript. JF performed most of experiments and all data analyses. HT performed some experiments in the early stage of study. All authors read and approved the submitted version.

## Conflict of Interest Statement

The authors declare that the research was conducted in the absence of any commercial or financial relationships that could be construed as a potential conflict of interest. The reviewer AM and handling Editor declared their shared affiliation.

## Abbreviations

AZLM: active-zone-like membrane
CAST: cytomatrix at the active zone-associated structural protein
CNQX: 6-cyano-7-nitroquinoxaline-2,3-dione
NND: nearest neighbor distance
PBS: phosphate buffered saline
SEP: super-ecliptic pHluorin
Syp: synaptophysin
TIRFM: total internal reflection fluorescence microscopy.
